# Water-soluble phosphorus contributes significantly to shaping the community structure of rhizospheric bacteria in rocky desertification areas

**DOI:** 10.1038/s41598-019-54943-z

**Published:** 2019-12-05

**Authors:** Jinge Xie, Wenzhi Xue, Cong Li, Zongqiang Yan, Dong Li, Guoqiang Li, Xiwen Chen, Defu Chen

**Affiliations:** 1 Department of Genetics and Cell Biology, College of Life Sciences, Nankai University, Tianjin, China; 2 Department of Biochemistry and Molecular Biology, College of Life Sciences, Nankai University, Tianjin, China; 3 Department of Microbiology, College of Life Sciences, Nankai University, Tianjin, China

**Keywords:** Ecology, Microbiology, Molecular biology, Ecology, Environmental sciences, Solid Earth sciences

## Abstract

Microorganisms play important roles in soil improvement. Therefore, clarifying the contribution of environmental factors in shaping the microbial community structure is beneficial to improve soil fertility in karst rocky desertification areas. Here, the bacterial community structures of eight rhizospheric soil samples collected from perennial fruit plantations were analysed using an Illumina HiSeq2500 platform. The diversity and abundance of bacteria in rocky desertification areas were significantly lower than those in non-rocky desertification areas, while the bacterial community structure was not significantly different between root surface and non-root surface soils in the same rhizospheric soil samples. Proteobacteria predominated in rocky desertification areas, while Actinobacteria predominated in non-rocky desertification areas. Correlation analysis revealed that water-soluble phosphorus content (r^2^ = 0.8258), latitude (r^2^ = 0.7556), altitude (r^2^ = 0.7501), and the age of fruit trees (r^2^ = 0.7321) were positively correlated with the bacterial community structure, while longitude, pH, and total phosphorus content did not significantly influence the soil bacterial community structure. As water-soluble phosphorus content is derived from insoluble phosphorus minerals, supplementing phosphorus-solubilising bacteria to soils in rocky desertification areas is a feasible strategy for accelerating the dissolution of insoluble phosphorus minerals and improving agricultural production and environment ecology.

## Introduction

According to the Second Rocky Desertification Monitoring Report of the State Forestry Administration, China still had about 120,000 km^2^ of rocky desertification areas in 2012^1^. There are a large number of rocky desertification areas in the southwest of China, mainly concentrated in the karst areas, known as karst rocky desertification areas. Rocky desertification areas have fragile ecological environments, poor soil quality, reduced productivity, and decreased levels of phosphorus, which is indispensable for plant growth and development^1–3^.

Phosphorus is a component of many products, such as nucleic acid, phospholipids, and adenosine triphosphate (ATP). Various metabolic processes, such as photosynthesis, respiration, and energy transfer, require phosphorus^4,5^. Therefore, phosphorus is an indispensable element for plant growth and development. To compensate for phosphorus deficiency in the soil of rocky desertification areas, a large amount of phosphorus fertilizer is used. However, the utilisation rate of phosphorus fertilizer is only 20–30%. A large amount of phosphorus is fixed by minerals or metal ions in soil^6^.

Moreover, accumulated phosphorus fertiliser can be washed away by rain, causing pollution to other soils and groundwater^7,8^, and eutrophication of rivers and lakes^9^. Therefore, it is of great significance to identify methods that can not only increase the available phosphorus content in soil to ensure agriculture production but also reduce environmental pollution for ecological protection. In rocky desertification areas, phosphorus is more easily fixed due to high calcium content^10^, so it is particularly important to increase the available phosphorus content in soil.

Soil microorganisms play an important role in soil nutrient cycling, productivity, and ecosytems^11,12^. For example, phosphorus-solubilising microorganisms can convert insoluble phosphate into soluble phosphorus^13^. Potassium bacteria, such as *Bacillus* strains^14^, can transform potassium-insoluble minerals into soluble forms which are absorbed and utilized by plants. Some plant growth-promoting microorganisms regulate the growth of plants through phytohormones. For instance, *Bacillus* sp. LZR216 can promote the polar auxin transport to regulate the development of root system architecture in *Arabidopsis* seedlings^15^. *Pseudomonas* spp. trigger lateral root formation and promote root hair growth via auxin signalling^16^. Nitrogen-fixing symbiotic relationships also exist between rhizobia and the roots of leguminous plants^17^. Therefore, the application of microorganisms for agricultural fertilization or the prevention of pests is an important biotechnological strategy.

Phosphorus-solubilising microorganisms include phosphorus-solubilising bacteria (PSB) and phosphorus-solubilising fungi (PSF). Compared with PSF, PSB are more widely distributed and comprehensively studied. Some PSB belong to Proteobacteria, such as *Pseudomonas*, *Rhizobium*, *Burkholderia*, and *Erwinia*^13^. It has been found that the abundance of bacteria that dissolve inorganic phosphorus increases with soil pH in the range of 4–8^18^. Comparisons of the population and quantity of PSB in the rhizospheres of peanuts, duck foot, sorghum, and corn revealed that the number of PSB in the peanut rhizosphere is the greatest, while that in corn crops is the least^19^. The addition of straw biochar can increase the abundance of the inorganic phosphate-solubilising bacterial community in soil^20^. The type and number of PSB appear to be affected by environmental factors, such as soil type, plant species, and the ecological environment. Therefore, understanding the structure of the bacterial community in rocky desertification areas and clarifying its dominant bacterial community is of great significance for the development and utilization of PSB. However, the composition of soil microorganisms is complex, and the soil microorganisms interact with the environmental system^21^. Soil physicochemical properties, such as pH, geographical environment and carbon, nitrogen, phosphorus, and potassium content, affect their microbial communities^22,23^. Contaminants can lead to the succession of soil microbial communities^24^, while some microorganisms can degrade pollutants and restore the environment^25^. Therefore, if appropriate PSBs, such as the high-efficiency phosphate-dissolving PSBs from local soil, are inoculated into the rocky desertification areas, they would not only effectively improve the available phosphorus content of the soil, but also prevent environmental pollution caused by fertilisation.

To improve agricultural production and the ecological environment, local governments in China encourage farmers to plant fruit trees with good economic benefits, barren tolerance, drought resistance, and well-developed roots in the severe rocky desertification areas, including *Amygdalus persica* L., *Armeniaca vulgaris* Lam., *Castanea mollissima* BL., *Citrus sinensis* L. Osbeck, *C*. *maxima* (Burm) Merr., *C. aurantium* L., and *Ziziphus jujuba* Mill. These fruit trees are perennial, usually grafted with fine varieties of scion on robust rootstocks. Meanwhile, previous studies have investigated the driving forces of rocky desertification^26^, changes in soil, space, and environment^27–29^, and the improvement of the ecological environment through vegetation restoration^30,31^. However, the microbial structure in fruit tree rhizosphere soils in rocky desertification areas remains unclear. Therefore, it is of great significance to understand the microbial structure and its relationship with environmental factors to promote the sustainable development of agricultural production and the ecological environment in rocky desertification areas using native bacterium.

In this study, we analysed the relationship between environmental factors (latitude, longitude, altitude, soil pH, total soil phosphorus content, water-soluble phosphorus soil content, and the age of fruit trees) and the bacterial community structures of planted fruit tree rhizosphere soils in rocky and non-rocky desertification areas using high-throughput sequencing of the 16 S V4 region. This study primarily explored (1) the differences in bacterial communities between the rocky and non-rocky desertification areas and (2) the effects of the environmental factors on the bacterial community structures.

## Results

### The pH and phosphorus content in fruit tree rhizosphere soils

To ensure the representativeness and comparability of the soil samples, six rocky desertification sampling sites and two non-rocky desertification sits were selected to determine the pH, as well as total phosphorus (TP) and water-soluble phosphorus (WSP) contents. The pH values in the rhizosphere soils differed significantly, ranging from 5.35–8.40 (Table 1). In the rocky desertification areas, the soils in KNY2 and KXQ2 were strongly acidic, KHT1 and KXB1 were weakly acidic, and KGY1 and KHZ1 were weakly alkaline. While in non-rocky desertification areas, soils in NCX2 and NCX3 were weakly alkaline.

**Table 1:** Locations, physicochemical properties, and planted fruit trees of the soil samples.

Soil samples		Locations				Physicochemical properties*			Planted fruit tree	
Type	Code	Latitude	Longitude	Altitude/m	County, province	pH	TP/g kg^−1^ DW	WSP/mg kg^−1^ DW	Species	Tree age/years
Karst	KGY1	28°01′25″N	108°29′07″E	517	Yinjiang, Guizhou	8.40 ± 0.01 A	0.58 ± 0.01 B	0.69 ± 0.20 D	*Citrus maxima* (Burm) Merr.	10
	KHT1	28°27′40″N	109°29″52″E	550	Huayuan, Hunan	6.84 ± 0.07 C	0.60 ± 0.05 B	0.20 ± 0.03D	*Amygdalus persica* L.	3
	KHZ1	28°26′33″N	109°28′49″E	550	Huayuan, Hunan	7.94 ± 0.02 B	0.31 ± 0.03 CD	0.20 ± 0.03 D	*Ziziphus jujuba* Mill.	3
	KNY2	28°12′38″N	112°35′42″E	388	Ningwu, Hunan	5.35 ± 0.10 F	0.29 ± 0.01 D	1.37 ± 0.39 C	*Citrus aurantium* L.	10
	KXB1	26°32′08″N	110°45′41″E	365	Xinning, Hunan	6.20 ± 0.06 D	0.37 ± 0.03 CD	0.29 ± 0.05 D	*Castanea mollissima* BL.	5
Non-karst	KXQ2	26°31′46″N	110°45′03″E	353	Xinning, Hunan	5.70 ± 0.16 E	0.55 ± 0.04 B	0.91 ± 0.19 CD	*Citrus sinensis* L. Osbeck	10
	NCX2	43°54′13″N	125°20′37″E	211	Changchun, Jilin	8.38 ± 0.14 A	0.71 ± 0.02 A	6.89 ± 0.21 A	*Armeniaca vulgaris* Lam.	20
	NCX3	43°49′13″N	125°16′41″E	251	Changchun, Jilin	8.27 ± 0.04 A	0.38 ± 0.03 C	4.71 ± 0.42B	*Armeniaca vulgaris* Lam.	10

WSP, the water-soluble phosphorus content; TP, the total phosphorus content. *, the data comparison experiments were performed in triplicate. Values are shown as means ± SD. Different capital letters indicate significant differences based on the S-N-K method of one-way ANOVA at *P* < 0.01 with IBM SPSS Statistics 22.0 (SPSS Inc., Chicago, IL, USA).

The TP content of eight rhizosphere soils was different, ranging from 0.29–0.71 g kg^−1^ DW (Table 1). However, no significant difference was detected between the rocky and non-rocky desertification areas (0.45 *vs*. 0.54 g kg^−1^ DW). In contrast, WSP content was significantly different among these soil samples (0.20–6.89 mg kg^−1^ DW), and WSP content in rocky desertification areas was approximately 10% that of non-rocky desertification areas. These data further confirm that the soil in rocky desertification areas is barren. The WSP content in the soil may be affected by several factors, such as pH value, calcium content, fruit trees species, and microbial abundance. Therefore, it is important to determine the microbial community structure and its correlation with environmental factors.

### Illumina HiSeq analysis of the bacterial 16 S V4 region

To analyse the bacterial community structure of fruit tree rhizosphere soils in rocky desertification areas, Illumina HiSeq analysis of the bacterial 16 S V4 region of soil samples from the root surface (S) and non-root surface (N) was performed. The effective tags ranged from 55791 to 92870 reads (average 79711.1 reads), accounting for 86.37–93.86% (average 90.76%) of raw reads (Supplemental Table S1). The average of effective tags was 253.1 bp (253–255 bp), and the GC content was 56.60% (55.24–57.42%). In effective tags, 99.36% (99.27–99.47%) of base quality met the Q20 standard (sequencing error rate less than 1%), and 98.69% (98.53–98.87%) met the Q30 standard (sequencing error rate less than 0.1%) (Supplemental Table S1). These data indicate that Illumina HiSeq sequencing was effective and could be used for subsequent bacterial community structure analyses. The rarefaction curve and Shannon-wiener curve (Supplemental Fig. S1) further confirmed that the sampling of the reads was sufficient and could accurately reflect the bacterial community in soil samples.

### OTU clustering and bacterial community analysis

**Figure 1:**
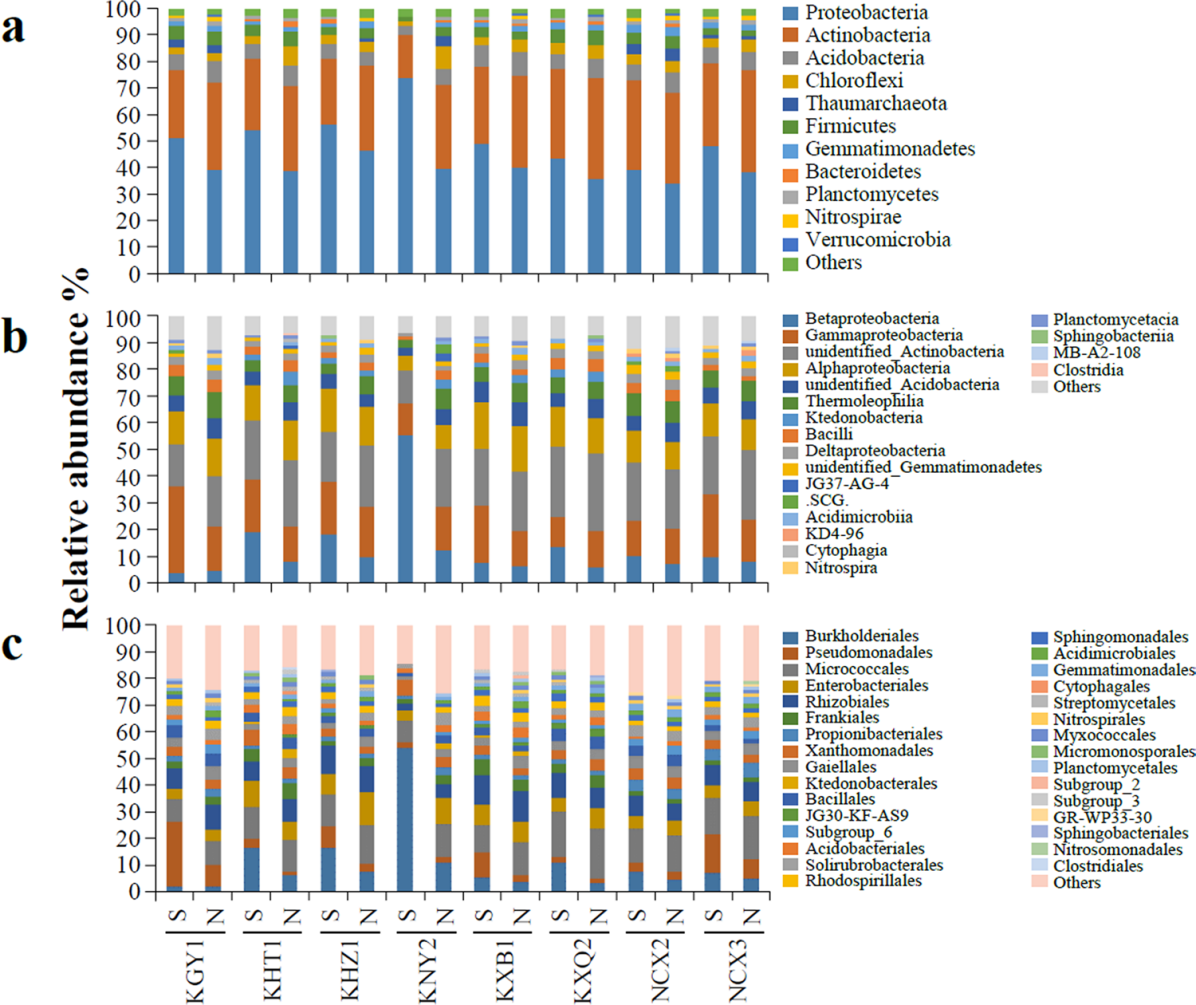
Relative abundances of the most abundant bacterial phyla (**a**), classes (**b**), and orders (**c**) in soil samples. S denotes root surface soil and N denotes non-root surface soil.

After obtaining the effective tags of the 16 S V4 region, OUT clustering was performed. The average number of OTUs was 5146 (4072–5656) in each soil sample (Table 2). Annotation of the representative OTU sequence found that 99.55% of bacteria belonged to 44 phyla. Among them, 11 phyla accounted for more than 1% of the total bacteria. However, only five phyla accounted for more than 1% of bacteria in all samples (Fig. 1a), and four phyla accounting for more than 1% were only present in the non-rocky desertification samples (Supplemental Table S2).

**Table 2:** General information of the soil bacterial high-throughput sequencing data.

Soil bacterial samples			OTU	Phylum	Class	Order	Family	Genus	Species
Type	Code	Soil species							
Karst	KGY1	S	4808	31	81	153	299	494	256
		N	5210	30	75	150	286	475	251
	KHT1	S	4590	33	74	154	289	471	265
		N	4873	36	81	156	292	471	260
	KHZ1	S	4952	33	80	155	285	471	269
		N	5143	32	75	149	285	468	266
	KNY2	S	4072	31	70	137	265	409	214
		N	5018	36	79	152	283	461	251
	KXB1	S	5371	34	85	164	306	515	277
		N	5306	36	80	154	293	463	256
	KXQ2	S	5441	32	82	158	297	495	282
		N	5613	35	83	162	301	512	296
Non-karst	NCX2	S	5550	30	81	153	292	469	259
		N	5656	38	82	151	294	478	258
	NCX3	S	5311	36	82	154	293	459	245
		N	5421	32	78	143	282	447	222
**Mean**			**5146**	**33**	**79**	**153**	**290**	**472**	**258**

Although the proportion of Proteobacteria and Actinobacteria in the soil samples varied, these two bacteria always had the highest proportion. Proteobacteria accounted for 45.40% (34.11–73.80%), while Actinobacteria accounted for 30.97% (16.32–38.33%) of the community. In rocky desertification areas, Proteobacteria accounted for 47.24% (35.58–73.80%) of the community, which was higher than that in the non-rocky desertification areas (39.89% (34.11–47.97%)); while Actinobacteria accounted for 29.84% (16.32–37.93%), which was lower than that in non-rocky desertification areas (34.35% (31.20–38.33%)). These data indicated that Proteobacteria and Actinobacteria may be the dominant bacteria in rocky or non-rocky desertification areas, respectively.

Species annotation revealed that 97.78% (96.22–99.35%) of the bacteria belonged to 99 classes. Of these, 20 classes accounted for more than 1% of the community (Fig. 1b). Of these 20 classes, eight classes were present in all soil samples, and three classes were present in all the samples from the non-rocky desertification areas (Supplemental Table S3). Further analysis revealed that four classes belonged to Proteobacteria, accounting for 45.27% of the bacterial community, and four classes belonged to Actinobacteria, accounting for 30.23% of the community.

Of the total bacteria, 93.37% (88.15–97.51%) belonged to 201 orders. Of these, 31 orders accounted for more than 1% (Fig. 1c), and 12 of these orders were present in all soil samples and seven orders were present in all the samples from the non-rocky desertification areas (Supplemental Table S4). Further analysis revealed that ten orders belonged to Proteobacteria, which accounted for 42.01% of the bacterial community, while four orders belonged to Actinobacteria, which accounted for 28.17% of the community.

### Bacterial community diversity analyses

To further understand the bacterial community structure, ANOSIM analyses were performed on the two different types of soil samples. No difference in the bacterial community structure was revealed between the root surface soil (“S”) and non-root surface soil (“N”) in the same rhizosphere soil, with *P* values greater than 0.05 (Supplemental Table S5). However, a significant difference in the bacterial community structure was revealed between the samples from rocky and non-rocky desertification areas, with *P* values less than 0.05, and R values greater than zero **(**Table 3).

**Table 3:** Differences between soil samples by an analysis of similarities (ANOSIM).

Type		Karst							Non-karst		
	Code	KGY1	KNY2	KHZ1	KXB1	KXQ2	KHT1	K	NCX2	NCX3	NK
Karst	KGY1	/	**0.9383**	**0.9222**	**0.8870**	**0.9724**	**1.0000**	/	**0.9093**	**0.8370**	**0.8531**
	KNY2	0.002	/	**0.6630**	**0.7889**	**0.7611**	**0.6481**	/	**0.7963**	**0.8815**	**0.9405**
	KHZ1	0.003	0.006	/	**0.4093**	**0.6352**	**0.7574**	/	**0.8611**	**0.9000**	**0.9492**
	KXB1	0.002	0.002	0.002	/	**0.6667**	**0.9185**	/	**0.9815**	**0.9389**	**0.9746**
	KXQ2	0.002	0.004	0.002	0.002	/	**0.9593**	/	**0.9870**	**1.0000**	**0.9520**
	KHT1	0.004	0.005	0.006	0.003	0.006	/	/	**1.0000**	**1.0000**	**1.0000**
	K	/	/	/	/	/	/	/	**0.1450**	**0.2532**	**0.2544**
Non-karst	NCX2	0.008	0.003	0.002	0.003	0.005	0.001	0.122	/	**0.9037**	/
	NCX3	0.004	0.005	0.003	0.004	0.003	0.004	0.035	0.005	/	/
	NK	0.001	0.001	0.001	0.001	0.001	0.001	0.005	/	/	/

K, the samples from the rocky desertification areas (karst areas); NK, the samples from non-rocky desertification areas (non-karst areas). Data in bold font is *R*-value and Data in normal font is *P*-value.

**Figure 2:**
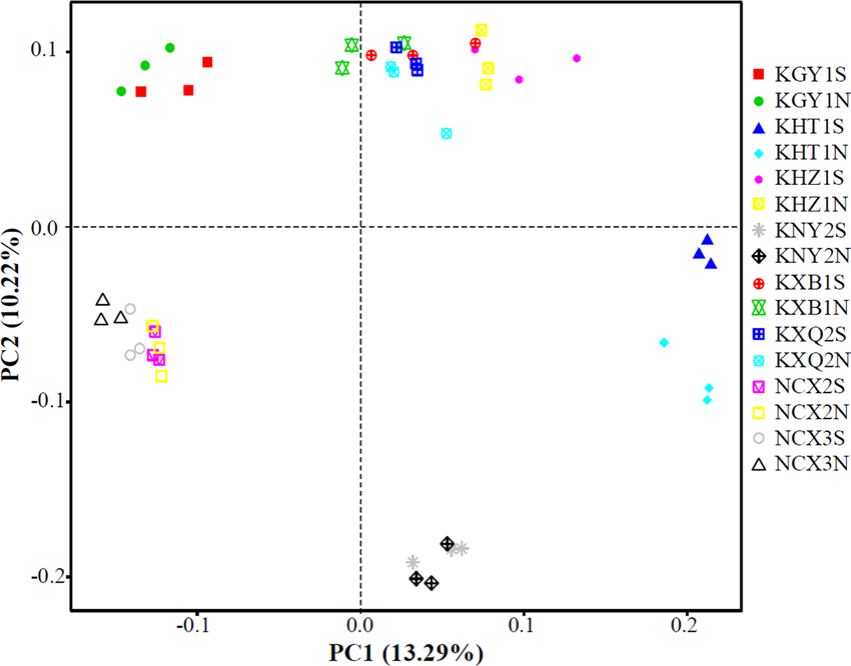
Principal coordinate analysis (PCoA) of the different soil bacterial community compositions based on the unweighted UniFrac distance metric.

**Figure 3:**
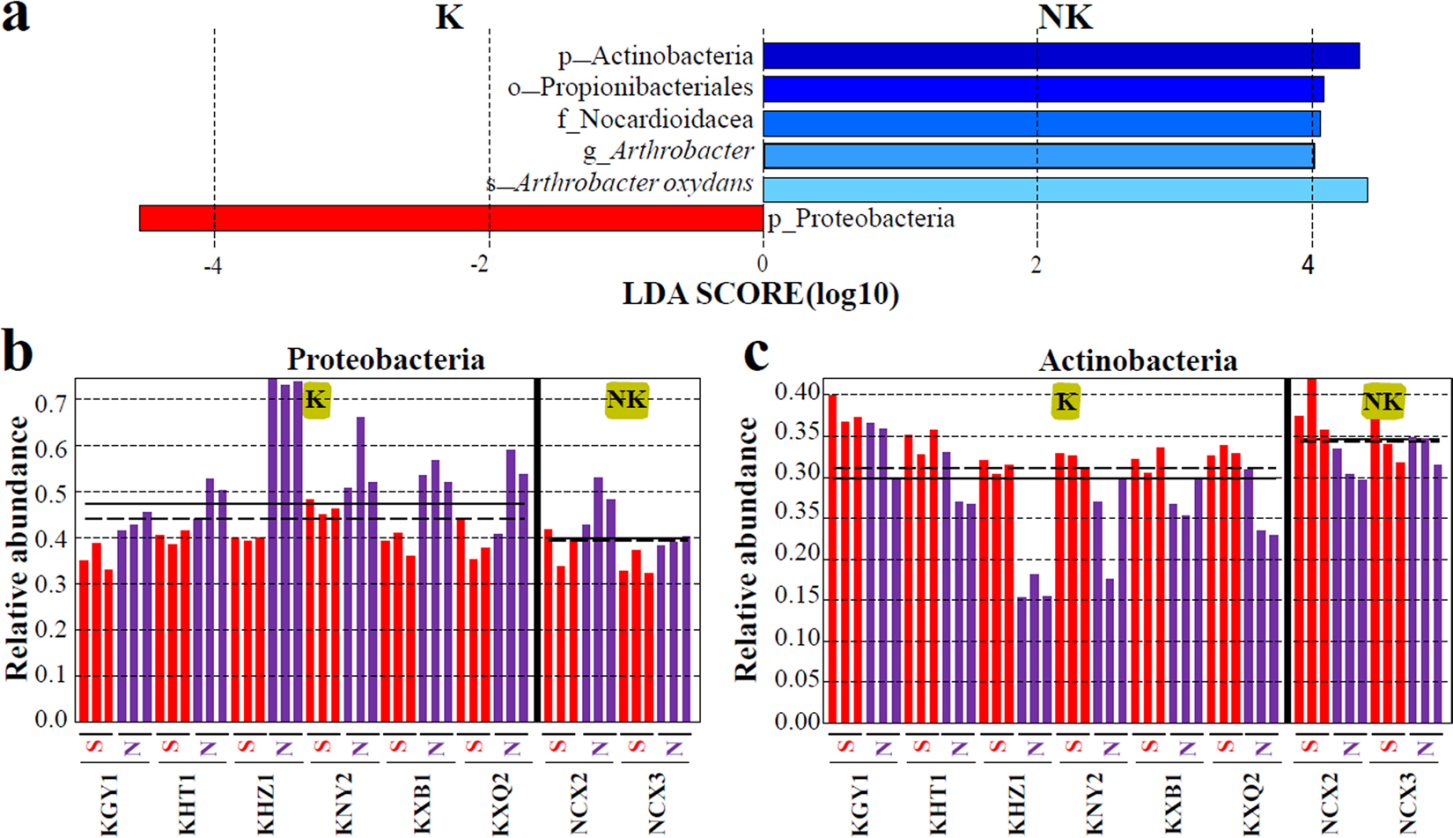
The significantly different bacteria in different soil samples. (**a**) linear discriminant analysis (LDA) effect size (LEfSe) analysis identifies phylum (p), class (c), order (o), family (f) genus (g) and species (s), which are significantly different among soil types, logarithmic LDA score ≥ 4.0. Proteobacteria (**b**) or Actinobacteria (**c**) biomarker abundance were compared in soil samples. K represents all the samples of rocky desertification areas (karst areas). NK represents all the samples of non-rocky desertification areas (non-karst areas).

To intuitively reflect the differences in bacterial community structure between the rocky and non-rocky desertification areas, PCoA analysis based on unweighted UniFrac distance was performed. As shown in Fig. 2, samples from rocky desertification areas and non-rocky desertification areas did not cluster. A linear discriminant analysis effect size (LEfSe) was performed to identify abundant differences in phylum, class, order, family, genus, and species between rocky and non-rocky desertification areas. No obvious dominant class, order, family, genus, or species were present in the rocky desertification areas. However, a dominant order (Propionibacteriales, average 4.25%), family (Nocardioidacea, average 4.03%), genus (*Arthrobacter*, average 11.59%), and species (*Arthrobacter oxydans*, average 9.92%) were present in the non-rocky desertification areas (Fig. 3a). At the phylum level, Proteobacteria was found to predominate in rocky desertification areas, while Actinobacteria predominated in the non-rocky desertification areas (Fig. 3a), which is consistent with the conclusions from the OTU cluster analyses. The abundance of Proteobacteria and Actinobacteria, shown in Fig. 3b,c, reflects the differences between the different soil samples.

Alpha diversity analysis of the bacterial community structure (Table 4) showed that the Shannon, Chao1, ACE, and PD indices of the soil samples in the rocky desertification areas were significantly lower than those for the non-rocky desertification areas. The observed species of the soil samples in the rocky desertification areas were significantly lower in number than for the non-rocky desertification areas. A diagram of petals with shared and unique OTUs was generated to compare the similarity and dissimilarity in bacterial community composition between the soil samples in rocky and non-rocky desertification areas (Supplemental Fig. S2). The average number of unique OTUs in rocky desertification areas (135) was lower than that in non-rocky desertification areas (180). These data indicate that the diversity and abundance of the bacterial community in the rocky desertification areas are lower than those in the non-rocky desertification areas.

**Table 4:** Alpha diversity indices of the soil bacterial samples.

Group	Observed species	Shannon	Chao1	ACE	PD	Good’s coverage
K	3370	8.26	4166	4334	194.2	0.9804
NK	3713	8.84	4549	4697	208.7	0.9790
*P* value	0.0037	0.0152	0.0120	0.0117	0.0048	0.1286

K, the samples from the rocky desertification areas (karst areas); NK, the samples from non-rocky desertification areas (non-karst areas). Observed species, Shannon, Chao1, ACE, PD and Good’s coverage were abbreviations of number of species observed, Shannon index, Chao 1 estimator, ACE estimator, phylogenetic diversity index and the index of sequencing depth. *P* value was based on the student’s *t*-test with IBM SPSS Statistics 22.0 (SPSS Inc., Chicago, IL, USA).

### Correlation between soil bacterial community and environmental factors

**Figure 4:**
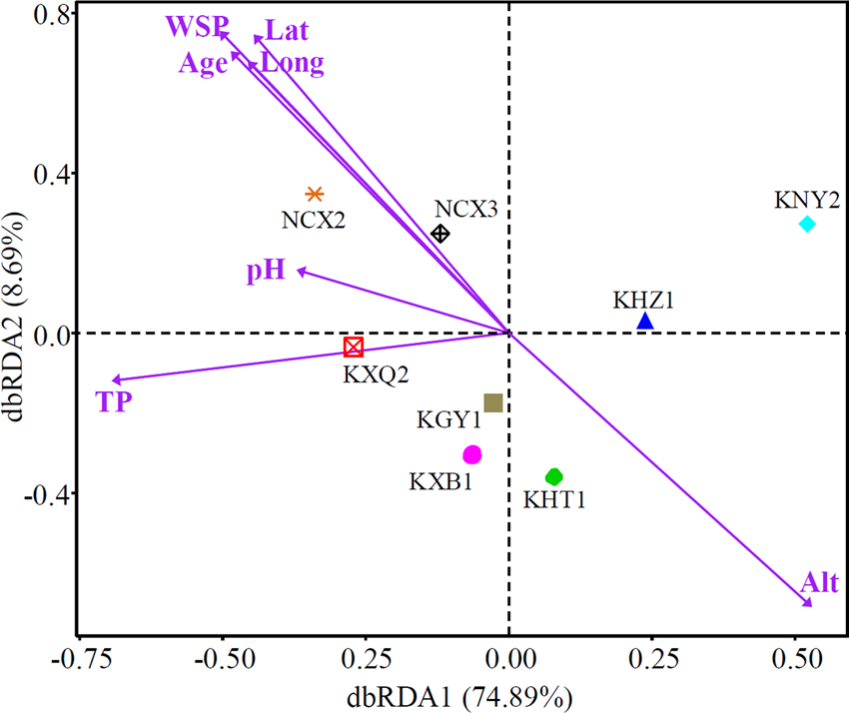
The correlation between the environmental factors and bacterial community structure by distance-based redundancy analysis (db-RDA). Lat, latitude; Long, longitude; Alt, altitude; pH, pH of the soil; WSP, water-soluble phosphorus content; TP, total phosphorus content; Age, the age of planting fruit trees.

Distance-based redundancy analysis (db-RDA) (Fig. 4) and a Monte Carlo permutation test (Table 5) were performed to examine the correlation between the environmental factors and soil bacterial communities. As shown in Fig. 4, the first and second axes explain 74.89% and 8.69% of the bacterial community values over phyla, respectively. The WSP content significantly influences the bacterial community (r^2^ = 0.8258, *P* = 0.024). The bacterial community structures in NCX2 and NCX3 were positively correlated with WSP, while those in KGY1, KNY2, KHZ1, KXB1, and KHT1 were negatively correlated with WSP. Latitude (r^2^ = 0.7556, *P* = 0.041), altitude (r^2^ = 0.7501, *P* = 0.026) and the age of fruit trees (r^2^ = 0.7321, *P* = 0.039) also significantly influenced the bacterial community structure. Longitude, pH, and TP did not significantly influence the soil bacterial community structure. As WSP is derived from insoluble phosphorus minerals, the solubility of soil phosphorus is crucial to soil fragility. Accelerating the dissolution of insoluble phosphorus minerals by PSB is of great significance for improvement the agricultural production and ecological environment in rocky desertification areas.

**Table 5:** The correlation between the environmental factors and soil bacterial community structure revealed by Monte Carlo permutation test.

Factor	RDA1	RDA2	r^2^	*P* value
Latitude	−0.5127	0.8586	0.7556	0.041
Longitude	−0.5574	0.8303	0.6697	0.076
Altitude /m	0.6115	−0.7912	0.7501	0.026
Soil pH	−0.9203	0.3913	0.1626	0.661
Soil TP /g kg^−1^ DW	−0.9855	−0.1699	0.4959	0.169
Soil WSP /mg kg^−1^ DW	−0.5561	0.8311	0.8258	0.024
Tree age /years	−0.5679	0.8231	0.7321	0.039

WSP, the water-soluble phosphorus content; TP, the total phosphorus content.

## Discussion

Microorganisms play important roles in soil improvement, and microbial community structure affects soil fertility and plant growth. In this study, high-throughput sequencing was performed to analyse the bacterial community structure of fruit tree rhizosphere soil in rocky and non-rocky desertification areas. Significant differences in bacterial community structures were revealed for different soil types. However, no significant difference in bacterial community structure was found between the root and non-root surfaces in the same rhizosphere soil (Figs. 1 and 2, and Supplemental Table S5). This result may be due to the environmental similarity in the root surface and non-root surface soil as the developed roots couple with the natural growth of fruit trees for many years. This conclusion is similar to that found by Xiao *et al*.^32^ in which the microbiomes in rhizosphere soil were similar to that in root zone soils at all levels (from genus to phylum).

In this study, significant differences in the bacterial community structures in the rhizosphere soils of rocky and non-rocky desertification areas were identified (Table 3). At the phylum level, Proteobacteria predominated in rocky desertification areas, while Actinobacteria predominated in non-rocky desertification areas (Fig. 3). It has been reported that Proteobacteria dissolve insoluble phosphorus minerals and participate in the phosphorus cycle^13,33,34^. Betaproteobacteria (belonging to Proteobacteria) are also reported to have a role in apatite dissolution and nutrient cycling^35^. Therefore, isolating these dominant bacteria and applying them back to the soil may be beneficial for improving soil quality.

Biogeographical and unique environmental conditions generally affect the distribution of Actinobacteria in soil^36^. In this study, it was found that the proportion of Actinobacteria (29.84% in rocky; 34.35% in non-rocky) was lower than that of Proteobacteria (47.2% in rocky; 39.89% in non-rocky) in both soil types, although Actinobacteria predominated in non-rocky desertification areas. This result could be attributed to the low adaptation of Actinobacteria to the harsh rocky desertification environment. Differences in the bacterial community structures of KHT1 and KNY2 from those in other rocky desertification areas suggest that other environmental factors may also affect the bacterial community structure of rhizosphere soil.

In this study, it was also revealed that the diversity and abundance of bacteria in rocky desertification areas is significantly lower than those in non-rocky desertification areas (Table 4 and Fig. S2). It has been reported that the degree of rocky soil desertification and vegetation restoration affects the physicochemical properties and then the growth of microorganisms and plants^3,30,37^. Therefore, it is quite logic that the soil fertility, particularly WSP content, affects the diversity and abundance of bacteria in rocky and non-rocky desertification areas.

Phosphorus is indispensable for plant growth and development, as well as for microorganisms. The relationship between available phosphorus and microbial community dynamics is complex and current knowledge regarding these mechanisms is incomplete^38^. In karst broadleaf forests, TP content has a greater effect on the microbial community than available phosphorus content^39^; while in the Black soil of northeast China, the microbial community structure is mainly affected by soil phosphorus, including both TP and available phosphorus, which are significantly increased by chemical fertilization^40^. Therefore, different forms of phosphorus have inconsistent effects on the microbial communities in different types of soil. In this study it was found that WSP content (r^2^ = 0.8258, *P* = 0.024) is the main factor affecting the bacterial community structure, while TP had no significant effect on the bacterial community structure (Fig. 4 and Table 5). These data indicate that WSP is an important indicator of soil fertility in rocky desertification areas, and the solubility of phosphorus is crucial for soil vulnerability. Although WSP is derived from insoluble phosphorus minerals, no linear relationship was found between WSP and TP in this study (Table 1). Therefore, supplementing PSBs to soil is an important measure to accelerate the dissolution of insoluble phosphorus minerals in soil and thus improve the agricultural production and ecological environment in rocky desertification areas.

The relationship between microbial structure and altitude is not considered to be significant^41^. However, the effect of altitude (r^2^ = 0.7501, *P* = 0.026) on the bacterial communities in this study was found to be significant (Fig. 4 and Table 5); therefore, this relationship may require further investigation. The correlation between latitude and bacterial community structure is not consistent. Some studies have previously reported that soil bacterial diversity is higher in low latitudes^42^, while others have reported that soil bacterial diversity has no^43^ or a parabolic relationship^44^ with latitude. In this study, it was found that latitude (r^2^ = 0.7556, *P* = 0.041) significantly affected the bacterial community, and the soil bacterial diversity was higher in high latitudes (Fig. 4 and Table 5). However, no significant influence of longitude on bacterial community structure was identified, which may be due to the minute difference in longitudes of the soil sampling areas. The growth and developmental stages of two cultivated legume plants have also been reported to affect the microbial community structures in rhizosphere soil^32^, which is consistent with the findings of the present study, i.e. the age of fruit trees (r^2^ = 0.7321, *P* = 0.039) significantly influenced the bacterial community. Bacteria grow effectively in a suitable pH; it has previously been reported that pH significantly affects the bacterial community structure in soil^11,45,46^. However, pH did not significantly affect the bacterial community structure in this study (Fig. 4 and Table 5), which is consistent with previous studies, such as those in Dongting Lake wetland^47^ and wetland soil^48^. In these studies, soil texture and heavy metals (Cd, Cr, Pb, and Cu) have greater effects on soil bacterial community structure than other physicochemical properties, such as pH value. The effect of pH value on the bacterial communities in these studies may have been masked by other prevailing factors, such as WSP, latitude, altitude, and the age of fruit trees.

In summary, the results of this study demonstrate that the bacterial community structures were significantly different between rocky and non-rocky desertification areas. Proteobacteria was the dominant phylum in the rocky desertification areas, while Actinobacteria was dominant in non-rocky desertification areas. No significant difference in bacterial community was found between the root surface and non-root surface in the same rhizosphere soil. WSP was found to be the primary factor shaping bacterial community structures in fruit tree rhizosphere soils. Latitude, altitude, and the age of fruit trees also had significant influences on the bacterial community structures in soils. Longitude, pH, and TP did not significantly influence the bacterial community structure in soils. These data contribute to the development of agricultural production and the restoration of ecological environments in rocky desertification areas from a microorganism aspect.

## Materials and Methods

### Collection of fruit tree rhizosphere soil samples

In August 2016, six sites (KGY1, KHT1, KHZ1, KNY2, KXB1, and KXQ2 (Table 1)) experiencing severe desertification were selected for sampling in the karst rocky desertification areas of southwestern China. Fruit trees that had not been affected by weeds, fertilizers, or pesticides for many years, had grown normally, and were producing healthy fruit were selected. At noon after several sunny days, the surface soil was removed and approximately 500 g of soil (10–20 cm depth) was retrieved and placed into sterile plastic bags with a sterile medicine spoon using the five-point sampling method. Due to the fruit trees with well-developed root systems, and the soils were very close, and almost attached to the roots, the soil samples were considered as “rhizosphere soils”. The samples were kept in a refrigerator at temperatures ranging from 4 °C to −70 °C. The sampling time, latitude, longitude, and altitude of the sampling sites, as well as species and age of fruit trees, were recorded. Meanwhile, the fruit tree rhizosphere soil in two sites (NCX2 and NCX3 (Table 1)) from the non-rocky desertification areas in northeast China were also selected for sampling, as described above.

### Determination of pH and phosphorus content in fruit tree rhizosphere soil

In total, 2 g of rhizosphere soil of the fruit trees was placed in a sterile Eppendorf tube then mixed with 5 mL of deionised water. pH values were determined using a pH meter (PH400, Alalis Instruments Technology Co., Ltd, Shanghai, China).

TP and WSP were extracted from the air-dried rhizosphere soil using the H_2_SO_4_-HClO_4_ method^49^, and the method described by Wu *et al*.^50^, respectively. The extraction of WSP was slightly modified. Briefly, 1 g of air-dried soil was placed in a 50 mL triangular flask. Then, 10 mL of sterile water was added. After shaking vigorously at 180 rpm with glass beads for 2 h at 25 °C, the extraction was filtered through non-phosphorus filter paper, and the obtained filtrate was WSP. The content of TP and WSP were determined according to the absorbance at a wavelength of 700 nm using the Mo-blue method^51^.

### Isolation of bacterial genomic DNA from fruit tree rhizosphere soil

The fruit tree roots in the rhizosphere soil were picked out. The soils that shaken gently into sterile tubes, were called non-root surface soil (called “N”). The remaining soils that attached firmly to the root surface, and were shaken strongly and swept carefully with sterile brushes into other sterile tubes, were called root surface soil (called “S”). Each soil sample (0.1 g) was placed in a sterile tube. After adding 4 mL of 1 × PBS buffer (137 mM NaCl, 2.7 mM KCl, 10 mM Na_2_HPO_4,_ and 2 mM KH_2_PO_4_, pH 7.4), the samples were shaken in a vortex mixer (Scilogex LLC., Rocky Hill, USA) at 2000 rpm for 20 min. The mixture was centrifuged twice at 200 rpm for 1 min to remove soil grain. The supernatant was centrifuged for 3 min at 12,000 rpm, and the precipitate was suspended in 2 mL 1 × PBS buffer. This procedure was repeated two-three times until the soil colour in the supernatants was not obvious. Then, 600 µL of Lysis buffer (50 mM Tris, 40 mM EDTA, and 10 mM NaCl, pH 8.0) was added to the precipitate to lyse the soil bacteria. The lysis solution was then transferred to a grinding tube containing 0.2 g of glass beads with a 0.1 mm diameter, and was ground for nine minutes in a biological sample homogenizer (Bioprep-24, Allsheng Instruments Co., Ltd., Hangzhou, China). Then, lysozyme at a final concentration of 10 mg mL^−1^ was added and incubated for 1 h at 37 °C followed by the addition of 120 µL of 20% SDS and incubation at 65 °C for 1–1.5 h. Next, extraction was undertaken using an AxyPrep Bacterial Genomic DNA Miniprep Kit (Axygen Scientific Inc., Silicon Valley, USA). The purity and concentration were detected using agarose gel electrophoresis. Once the quality was determined, the sample was diluted to 1 ng μL^−1^.

### Illumina HiSeq sequencing of the bacterial 16 S V4 region and acquisition of effective 16 S V4 tags

The 16 S V4 region was amplified using 515 F (5′-GTGCCAGCMGCCGCGGTAA-3′) and 806 R (5′-GGACTACHVGGGTWTCTAAT-3′) with the barcode as primers, and the isolated genomic DNA from the fruit tree rhizosphere soils as the template. Reactions were carried out using Phusion High-Fidelity PCR Master Mix (New England Biolabs, Inc., MA, USA). The product was displayed using 2% agarose gel electrophoresis, and the target band was collected using the QIAquick Gel Extraction Kit (QIAGEN Co., Ltd., Hilden, Germany). Sequencing libraries were generated using a TruSeq DNA PCR-Free Sample Preparation Kit (Illumina Inc., San Diego, California, USA). After being confirmed by Qubit and q-PCR, the library was sequenced on an Illumina HiSeq2500 platform. Paired-end reads were assigned to samples and merged into raw tags using FLASH^52^. All raw sequencing data were submitted to NCBI under the Bioproject accession number PRJNA555660. Quality filtering on the raw tags was performed under specific filtering conditions^53^ to obtain high-quality clean tags according to the QIIME quality-controlled process^54^. The tags were compared with the Gold database^55^ using the UCHIME algorithm^56^ to remove chimera sequences. Then, the effective tags were finally obtained.

### OTU cluster and species annotation

Sequence analyses were performed using the effective tags of the 16 S V4 region using Uparse software^57^. Sequences with ≥ 97% similarity were assigned to the same OTUs. The representative sequence for each OTU was screened for further annotation. Based on the RDP classifier algorithm^58^, the GreenGene Database^59^ was used to annotate the taxonomic information of each representative sequence. OTU abundance information was normalized using a standard of the sample with the least sequences. Alpha diversity and beta diversity were subsequently performed based on this output normalized data. A rarefaction curve and Shannon-Wiener curve of each sample were calculated using QIIME software, and displayed with R software (ver. 2.15.3). According to the results of the OTU clustering analysis, the shared and unique OTUs among different samples were analysed, and the petal diagram was plotted.

### Statistical analyses

A one-way analysis of variance (one-way ANOVA) was performed using IBM SPSS Statistics 22.0 (SPSS Inc., Chicago, IL, USA) to determine the differences in soil properties. An analysis of similarities (ANOSIM) was performed using the anosim function of the R vegan package to determine whether different soil samples had significantly different bacterial communities^60^. A principal coordinate analysis (PCoA) was performed using WGCNA, stat, and gg plot2 packages in R software (ver. 2.15.3) to demonstrate the relationships between different soil samples^54^. UniFrac distance was analysed using QIIME ver. 1.7.0 software. The significantly different bacteria communities in different soil types were analysed using linear discriminant analysis (LDA) effect size (LEfSe) using LEfSe software with the LDA score setting as 4^61^. Alpha diversity analysed the complexity of species diversity for a sample through six indices; observed species, Chao1, Shannon, ACE, good coverage, and PD_whole_tree. These indices were calculated for each sample using QIIME (ver. 1.7.0). The significance of alpha diversity was determined using Student’s *t*-test. The relevance of the environmental factors in explaining the distribution patterns of bacterial communities in different soil samples was conducted through distance-based redundancy analysis (db-RDA) using R software^60^.

## Supplementary information

Supplementary Information
