# Supplementary material for: Water-soluble phosphorus contributes significantly to shaping the community structure of rhizospheric bacteria in rocky desertification areas
Source: Sci Rep. 2019 Dec 5;9:18408. doi: 10.1038/s41598-019-54943-z (PMC6895182; doi:10.1038/s41598-019-54943-z)
Supplement: Supplementary file 1 — Supplementary Information [file 41598_2019_54943_MOESM1_ESM.pdf]

Supplementary Data

Water-soluble phosphorus contributes significantly to shaping the community structure of rhizospheric bacteria in rocky desertification areas

Jinge Xie[1], Wenzhi Xue[2], Cong Li[1], Zongqiang Yan[1], Dong Li[2], Guoqiang Li[3], Xiwen Chen[2] & Defu Chen[1]

[1] Department of Genetics and Cell Biology, College of Life Sciences, Nankai University, Tianjin, China.

[2] Department of Biochemistry and Molecular Biology, College of Life Sciences, Nankai University, Tianjin, China.

[3] Department of Microbiology, College of Life Sciences, Nankai University, Tianjin, China.

Correspondence and requests for materials should be addressed to X.C. (email: xiwenchen@nankai.edu.cn) and / or D.C. (email: chendefu@nankai.edu.cn)

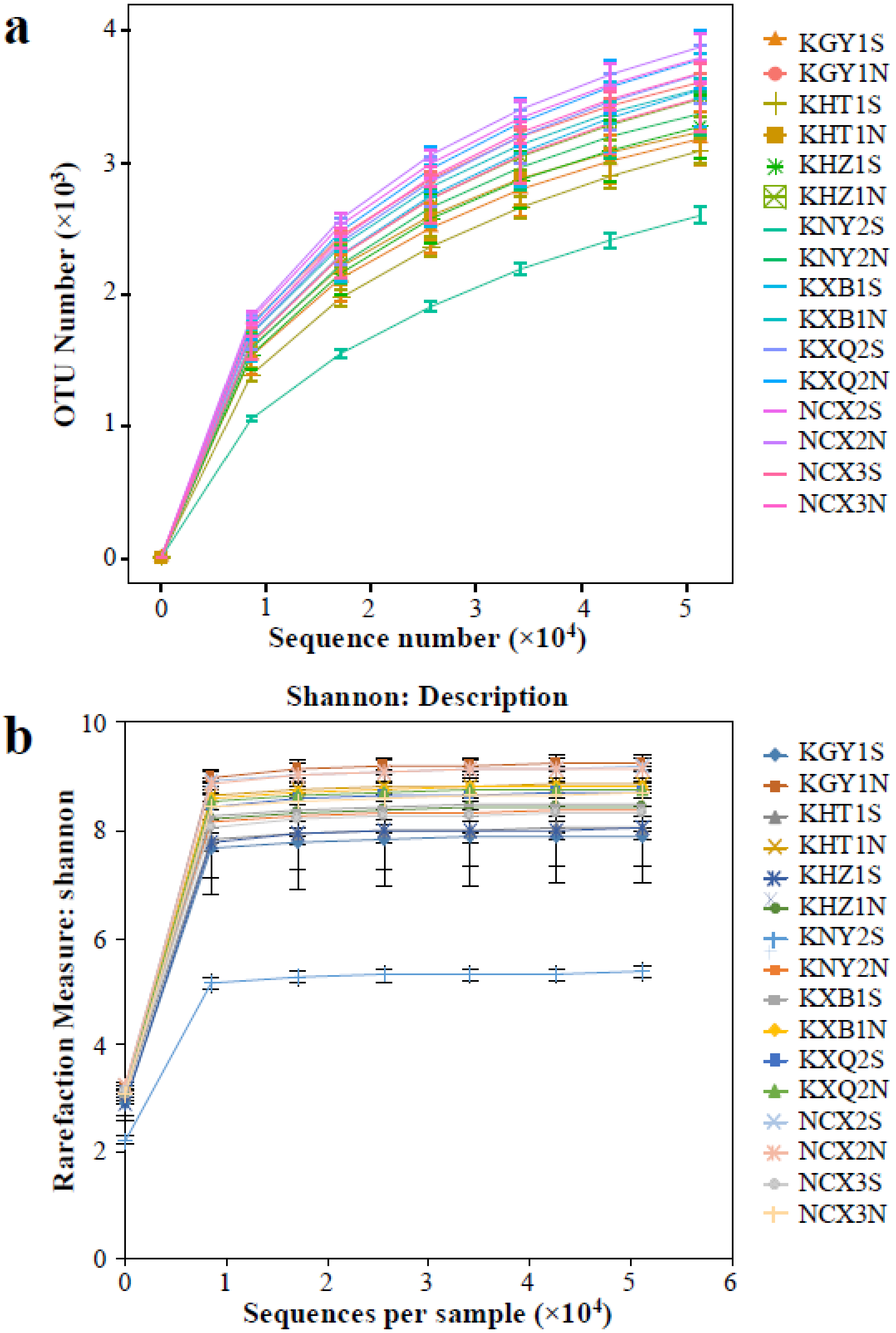

Figure S1. Rarefaction curve (**a**) and Shannon-Wiener curve (**b**) of the soil bacterial 16S V4 library.

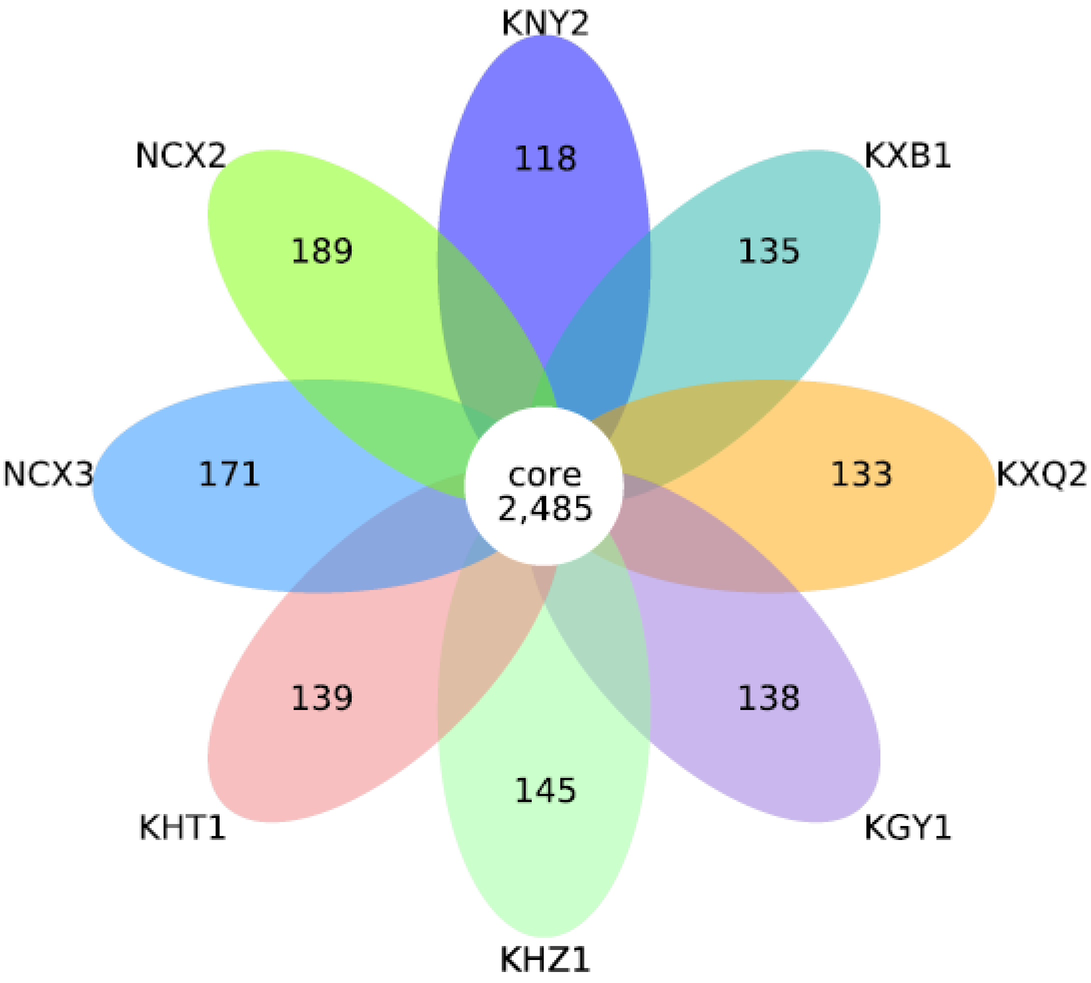

Figure S2. The diagram of petals with shared and unique OTUs in rocky and non-rocky desertification areas. The middle core number represents the number of shared OTUs in the all samples, and the number on the petals represents the number of OTUs in the sample.

Table S1. Statistics and quality control of Illumina HiSeq data pre-processing of the soil bacteria.

Sample	Raw PE	Raw tags	Clean tags	Effective tags	Effective rate %	Base	AvgLen	GC content %	Q20	Q30
KGY1S.1	93719	92260	91515	86256	92.04	21820407	253	55.82	99.47	98.87
KGY1S.2	80020	78597	77953	73320	91.63	18546487	253	55.24	99.43	98.82
KGY1S.3	84682	83224	82555	78316	92.48	19819330	253	56.65	99.43	98.82
KGY1N.1	88304	86688	85930	81047	91.78	20503455	253	56.91	99.42	98.79
KGY1N.2	91671	89820	89059	82599	90.10	20897591	253	56.91	99.41	98.78
KGY1N.3	86548	84873	84053	79116	91.41	20014656	253	56.57	99.40	98.76
KHT1S.1	95311	93587	92923	87013	91.29	22007923	253	56.50	99.43	98.81
KHT1S.2	95637	90304	88997	82598	86.37	21074970	255	56.04	99.27	98.53
KHT1S.3	93528	91683	90935	85463	91.38	21592382	253	56.18	99.34	98.66
KHT1N.1	64755	63574	62925	59512	91.90	15058998	253	56.68	99.32	98.62
KHT1N.2	86095	84477	83711	78637	91.34	19891079	253	56.82	99.34	98.65
KHT1N.3	61971	60617	60074	55791	90.03	14115998	253	56.69	99.32	98.64
KHZ1S.1	90362	88812	88107	83032	91.89	21005234	253	56.20	99.39	98.76
KHZ1S.2	72481	71172	70542	67121	92.60	16979416	253	55.82	99.29	98.59
KHZ1S.3	83252	81576	80826	74286	89.23	18802160	253	56.26	99.30	98.60
KHZ1N.1	99773	97926	96949	92870	93.08	23508942	253	56.70	99.27	98.54
KHZ1N.2	90572	88971	88169	82563	91.16	20899318	253	56.96	99.31	98.63
KHZ1N.3	80268	78840	78191	72324	90.10	18298615	253	56.91	99.40	98.75
KNY2S.1	97666	96183	95427	91279	93.46	23087739	253	56.02	99.39	98.74
KNY2S.2	89135	87413	86575	82204	92.22	20821490	253	55.84	99.37	98.73
KNY2S.3	97796	96152	95360	91790	93.86	23210242	253	55.80	99.41	98.81
KNY2N.1	87230	85632	84645	79583	91.23	20133933	253	56.90	99.32	98.64
KNY2N.2	84110	82350	81599	76628	91.10	19388824	253	56.98	99.34	98.68
KNY2N.3	85472	83676	82796	77158	90.27	19524278	253	56.83	99.29	98.60
KXB1S.1	93768	92307	91531	86026	91.74	21761821	253	56.60	99.38	98.73
KXB1S.2	99442	96951	95969	89249	89.75	22659519	254	55.89	99.30	98.60
KXB1S.3	91544	89909	89107	84661	92.48	21420556	253	56.92	99.35	98.65
KXB1N.1	80242	78832	78213	73925	92.13	18709232	253	56.78	99.33	98.64
KXB1N.2	84006	82362	81680	76055	90.54	19242252	253	56.93	99.36	98.67
KXB1N.3	82125	80993	80246	73398	89.37	18569958	253	57.36	99.40	98.74
KXQ2S.1	96493	94727	94002	87026	90.19	22016675	253	56.72	99.39	98.74
KXQ2S.2	86581	84991	84183	78920	91.15	20029642	254	56.75	99.41	98.76
KXQ2S.3	91507	89869	89058	82900	90.59	20971578	253	56.89	99.31	98.61
KXQ2N.1	89912	88210	87400	79277	88.17	20058855	253	57.42	99.30	98.59
KXQ2N.2	96238	94262	93414	86897	90.29	21984869	253	56.83	99.33	98.65
KXQ2N.3	87459	85836	85041	80256	91.76	20307605	253	56.79	99.34	98.64
NCX2S.1	87,925	86066	85191	79062	89.92	20004744	253	56.87	99.33	98.63
NCX2S.2	95001	93105	92090	86252	90.79	21872776	254	56.92	99.41	98.76
NCX2S.3	83103	81387	80567	73233	88.12	18526955	253	56.88	99.31	98.63

NCX2N.1	81481	78227	77251	71710	88.01	18172521	253	56.92	99.35	98.69
NCX2N.2	83959	82280	81522	75355	89.75	19068465	253	56.96	99.42	98.79
NCX2N.3	92798	90859	89961	85126	91.73	21569817	253	56.90	99.33	98.66
NCX3S.1	85314	83665	82885	75706	88.74	19153461	253	56.39	99.38	98.75
NCX3S.2	81532	80161	79490	72027	88.34	18221273	253	55.91	99.42	98.80
NCX3S.3	97257	95403	94529	89651	92.18	22681726	253	56.55	99.35	98.68
NCX3N.1	83595	82005	81202	75684	90.54	19164421	253	56.85	99.36	98.69
NCX3N.2	98879	97171	96328	86305	87.28	21833054	253	56.97	99.38	98.73
NCX3N.3	84386	82695	81841	76924	91.16	19466922	253	56.64	99.32	98.64

Raw PE, PE reads of the original offline machine; Raw Tags, tags sequence obtained by splicing; Clean Tags, the low-quality and short-length tags filtered; Effective Tags, the tags sequences that are finally used for subsequent analysis after filtering chimera; Effective rate, the percentage of effective tags to Raw PE; Base, the number of bases finally effective data; AvgLen, the average length of effective tags; GC content, the content of GC bases in effective tags; Q20 or Q30, the percentage of bases with the base mass value greater than 20 (sequencing error rate less than 1%) or 30 (sequencing error rate less than 0.1%) in effective tags.

Table S2. The relative abundance of the phyla of soil bacteria, which accounted for more than 1% in soil samples.

Phylum	Karst															Non-karst							Average		
	KGY1		KHT1		KHZ1		KNY2		KXB1		KXQ2		Average			NCX2		NCX3		Average					
	S	N	S	N	S	N	S	N	S	N	S	N	S	N	All	S	N	S	N	S	N	All	S	N	All
Proteobacteria	51.11	39.05	54.00	38.74	56.18	46.49	73.80	39.68	48.96	40.02	43.23	35.58	54.54	39.93	47.24	39.11	34.11	47.97	38.35	43.54	36.23	39.89	51.79	39.00	45.40
Actinobacteria	25.74	33.09	27.16	32.11	24.74	32.13	16.32	31.30	28.96	34.57	34.06	37.93	26.16	33.52	29.84	33.66	34.20	31.20	38.33	32.43	36.27	34.35	27.73	34.21	30.97
Acidobacteria	5.99	7.86	5.39	7.53	5.58	4.97	3.12	6.24	8.04	9.16	5.39	7.47	5.58	7.20	6.39	5.95	7.65	6.24	6.90	6.09	7.28	6.69	5.71	7.22	6.47
Chloroflexi	2.25	3.06	3.07	7.23	3.32	3.62	1.81	8.38	3.15	4.48	4.14	4.94	2.96	5.28	4.12	4.09	4.08	3.23	4.70	3.66	4.39	4.03	3.13	5.06	4.10
Firmicutes	5.11	5.31	4.16	5.77	3.00	3.73	1.84	3.73	4.00	2.96	5.31	5.60	3.90	4.52	4.21	4.39	4.84	2.52	2.12	3.45	3.48	3.47	3.79	4.26	4.02
Gemmatimonadetes	1.55	2.09	1.26	1.68	1.52	2.40	0.75	1.41	1.44	2.21	2.42	2.34	1.49	2.02	1.76	3.15	3.21	2.03	2.51	2.59	2.86	2.72	1.76	2.23	2.00
Thaumarchaeota	3.26	2.89	0.19	0.30	0.40	1.66	0.08	3.85	0.22	0.30	0.57	0.19	0.79	1.53	1.16	3.53	4.84	1.44	1.10	2.49	2.97	2.73	1.21	1.89	1.55
Planctomycetes	1.29	1.92	1.13	1.36	1.13	0.80	0.52	1.38	1.30	1.75	1.01	1.43	1.07	1.44	1.25	1.25	1.55	1.13	1.63	1.19	1.59	1.39	1.10	1.48	1.29
Bacteroidetes	0.74	0.69	1.11	2.16	1.53	0.67	0.40	0.76	1.00	0.67	1.02	1.33	0.97	1.05	1.01	0.89	1.18	0.76	0.59	0.83	0.89	0.86	0.93	1.01	0.97
Nitrospirae	1.02	1.50	0.31	0.43	0.59	1.47	0.33	0.57	0.71	1.23	0.74	0.73	0.62	0.99	0.80	1.39	1.45	1.15	1.39	1.27	1.42	1.34	0.78	1.10	0.94
Verrucomicrobia	0.75	1.06	0.79	0.89	0.93	0.80	0.35	0.73	1.00	1.16	0.80	1.01	0.77	0.94	0.86	0.92	1.19	0.90	0.89	0.91	1.04	0.97	0.80	0.97	0.88
Others	1.19	1.49	1.42	1.80	1.08	1.26	0.68	1.98	1.22	1.50	1.32	1.45	1.15	1.58	1.37	1.67	1.70	1.43	1.49	1.55	1.60	1.57	1.25	1.59	1.42

S, the root surface soil; N, the non-root surface soil.

Table S3. The relative abundance of the class of soil bacterium, which accounted for more than 1% in soil samples.

Taxonomy		Karst												Non-karst			
Phylum	Class	KGY1		KHT1		KHZ1		KNY2		KXB1		KXQ2		NCX2		NCX3	
		S	N	S	N	S	N	S	N	S	N	S	N	S	N	S	N
Actinobacteria	unidentified_Actinobacteria	15.94	18.77	21.92	24.50	18.84	22.86	12.29	21.70	21.28	22.17	26.04	29.11	21.43	22.35	21.81	26.00
	Thermoleophilia	7.04	9.63	4.20	6.08	3.93	6.55	3.04	7.41	5.44	7.30	5.83	6.56	8.54	8.01	6.29	7.84
	Acidimicrobiia	1.63	2.46	0.76	1.18	1.07	1.57	0.55	1.32	1.42	2.27	1.38	1.33	1.83	1.76	1.56	1.99
	MB-A2-108	0.44	0.89	0.09	0.12	0.42	0.57	0.24	0.46	0.28	0.58	0.37	0.42	1.00	1.17	0.70	1.10
Proteobacteria	Gammaproteobacteria	32.22	16.49	19.56	13.17	19.54	18.74	12.29	16.18	21.09	13.35	11.44	13.72	13.21	13.17	23.32	15.89
	Alphaproteobacteria	12.27	14.31	13.22	14.72	16.03	14.66	5.34	8.99	17.34	17.04	15.20	13.08	11.97	10.28	12.31	11.57
	Betaproteobacteria	3.82	4.55	19.11	8.18	18.24	9.67	54.99	12.32	7.70	6.15	13.31	5.70	10.26	6.97	9.60	7.86
	Deltaproteobacteria	2.70	3.59	1.94	2.45	2.23	3.24	1.12	2.03	2.64	3.31	3.13	2.88	3.60	3.61	2.66	2.95
Acidobacteria	unidentified_Acidobacteria	5.81	7.59	5.10	7.14	5.36	4.60	2.97	5.95	7.75	8.87	5.03	7.08	5.48	7.21	5.92	6.53
Firmicutes	Bacilli	4.54	4.63	3.19	4.56	2.33	3.00	1.44	3.06	3.35	2.42	4.58	4.77	3.83	4.21	2.18	1.76
	Clostridia	0.52	0.60	0.85	1.04	0.56	0.64	0.36	0.55	0.57	0.48	0.64	0.72	0.48	0.53	0.28	0.31
Gemmatimonadetes	unidentified_Gemmatimonadetes	1.55	2.09	1.26	1.68	1.52	2.40	0.75	1.41	1.44	2.21	2.42	2.34	3.15	3.21	2.03	2.51
Chloroflexi	Ktedonobacteria	0.62	0.94	2.14	5.02	2.08	2.12	0.77	3.65	1.74	2.61	2.84	3.60	0.95	0.99	0.67	0.81
	KD4-96	0.43	0.97	0.24	0.25	0.34	0.37	0.25	0.50	0.34	0.66	0.46	0.46	1.38	1.35	0.90	1.98
	JG37-AG-4	0.04	0.04	0.19	1.16	0.09	0.10	0.45	3.25	0.15	0.12	0.07	0.10	0.48	0.52	0.46	0.44
Planctomycetes	Planctomycetacia	1.09	1.54	1.03	1.24	1.00	0.68	0.47	1.25	1.15	1.56	0.85	1.22	0.98	1.23	0.91	1.28
Nitrospirae	Nitrospira	1.02	1.50	0.31	0.43	0.59	1.47	0.33	0.57	0.71	1.23	0.74	0.73	1.39	1.45	1.15	1.39
Thaumarchaeota	.SCG.	1.27	0.92	0.11	0.17	0.24	0.61	0.06	3.26	0.12	0.13	0.44	0.10	1.30	2.02	0.64	0.48
Bacteroidetes	Sphingobacteriia	0.51	0.53	0.67	0.77	1.23	0.50	0.28	0.43	0.64	0.47	0.75	1.02	0.67	0.93	0.50	0.42
	Cytophagia	0.16	0.14	0.42	1.36	0.22	0.14	0.11	0.31	0.25	0.16	0.21	0.26	0.18	0.20	0.21	0.14
Others		6.91	7.83	3.69	4.80	4.12	5.50	1.93	5.39	4.61	6.89	4.28	4.80	7.90	8.85	5.89	6.75

S, the root surface soil; N, the non-root surface soil.

Table S4. The relative abundance of the order in soil bacterium, which accounted for more than 1% in soil samples.

Taxonomy			Karst												Non-karst			
Phylum	Class	Order	KGY1		KHT1		KHZ1		KNY2		KXB1		KXQ2		NCX2		NCX3	
			S	N	S	N	S	N	S	N	S	N	S	N	S	N	S	N
Actinobacteria	unidentified_Actinobacteria	Micrococcales	8.35	8.84	11.75	11.61	11.67	14.40	7.95	12.56	10.02	12.30	17.05	18.89	12.41	13.87	13.56	16.14
		Frankiales	2.46	2.79	4.45	5.93	1.92	2.70	1.52	3.58	6.15	4.37	3.44	4.91	1.92	2.00	1.69	1.70
		Propionibacteriales	1.98	3.03	1.50	1.83	1.53	1.68	1.22	2.74	1.61	1.82	1.76	1.69	4.09	3.53	4.00	5.40
		Streptomycetales	0.85	1.43	1.45	1.79	1.33	1.26	0.47	0.84	1.06	1.21	1.12	0.99	0.82	0.71	0.74	0.93
		Micromonosporales	0.70	0.81	1.10	1.73	0.79	1.33	0.29	0.70	0.92	1.05	1.15	1.02	0.67	0.58	0.50	0.45
	Thermoleophilia	Gaiellales	3.60	5.34	2.03	3.07	2.25	3.90	1.21	2.90	3.10	4.80	3.39	3.93	4.41	4.12	3.10	4.11
		Solirubrobacterales	3.43	4.29	2.17	3.00	1.68	2.65	1.83	4.50	2.33	2.49	2.43	2.62	4.12	3.88	3.19	3.73
	Acidimicrobiia	Acidimicrobiales	1.63	2.46	0.76	1.18	1.07	1.57	0.55	1.32	1.42	2.27	1.38	1.33	1.83	1.76	1.56	1.99
Proteobacteria	Betaproteobacteria	Burkholderiales	2.14	2.11	16.70	6.30	16.36	7.40	53.83	11.04	5.56	3.62	10.97	3.48	7.69	4.75	7.33	5.25
		Nitrosomonadales	0.58	0.86	0.52	0.55	0.51	0.71	0.33	0.43	0.62	0.82	0.71	0.69	0.93	0.78	0.86	1.17
	Alphaproteobacteria	Rhizobiales	7.60	9.37	7.12	8.45	10.60	9.75	2.68	4.83	10.95	11.45	9.07	7.46	7.51	6.22	7.71	7.54
		Rhodospirillales	2.73	2.86	2.47	3.66	2.36	2.67	0.96	2.47	3.50	3.23	2.46	2.92	1.75	1.82	2.19	1.89
		Sphingomonadales	1.37	1.47	2.26	1.82	2.34	1.69	0.93	1.08	2.14	1.75	2.89	2.11	2.04	1.70	1.71	1.57
	Gammaproteobacteria	Enterobacteriales	4.08	4.28	10.06	6.84	7.70	12.39	3.83	9.89	7.71	7.42	5.07	7.54	4.88	5.39	4.84	5.36
		Pseudomonadales	24.26	8.20	3.23	1.41	8.37	3.31	2.25	1.90	9.42	2.90	2.31	1.50	3.47	2.73	14.22	7.09
		Xanthomonadales	3.27	3.24	5.84	4.50	3.01	2.53	6.00	4.05	3.28	2.47	3.57	4.20	4.37	4.54	3.65	2.86
	Deltaproteobacteria	Myxococcales	1.27	1.68	1.35	1.59	1.34	1.69	0.54	0.91	1.45	1.69	1.77	1.51	1.49	1.34	1.10	1.25
		GR-WP33-30	0.63	0.95	0.32	0.49	0.46	0.90	0.39	0.81	0.59	0.87	0.69	0.70	1.22	1.36	0.92	1.03
Firmicutes	Bacilli	Bacillales	4.49	4.62	3.18	4.50	2.32	2.98	1.43	3.02	3.30	2.40	4.56	4.75	3.82	4.18	2.14	1.76
	Clostridia	Clostridiales	0.52	0.59	0.85	1.03	0.56	0.64	0.36	0.54	0.57	0.48	0.63	0.71	0.47	0.53	0.28	0.31
Acidobacteria	unidentified_Acidobacteria	Acidobacteriales	1.61	1.89	2.86	3.72	1.84	1.81	1.53	2.58	3.40	3.73	1.89	2.96	1.02	1.58	1.51	1.38
		Subgroup_6	2.38	3.21	0.58	0.73	1.66	0.93	0.69	1.21	1.33	1.55	1.19	1.63	2.63	3.54	2.40	2.90
		Subgroup_3	0.64	0.62	0.92	1.50	0.82	0.82	0.28	0.77	1.36	1.10	1.02	0.99	0.53	0.48	0.58	0.46
		Subgroup_2	0.27	0.36	0.39	0.79	0.31	0.27	0.19	0.86	0.86	1.14	0.28	0.60	0.16	0.24	0.40	0.39
Gemmatimonadetes	unidentified_Gemmatimonadetes	Gemmatimonadales	1.15	1.64	1.19	1.58	1.46	2.32	0.69	1.32	1.34	2.08	1.88	1.91	2.51	2.67	1.87	2.30
Planctomycetes	Planctomycetacia	Planctomycetales	1.09	1.54	1.03	1.24	1.00	0.68	0.47	1.25	1.15	1.56	0.85	1.22	0.98	1.23	0.91	1.28
Nitrospirae	Nitrospira	Nitrospirales	1.02	1.50	0.31	0.43	0.59	1.47	0.33	0.57	0.71	1.23	0.74	0.73	1.39	1.45	1.15	1.39
Chloroflexi	Ktedonobacteria	JG30-KF-AS9	0.35	0.41	0.83	1.08	1.41	1.44	0.17	0.41	1.01	1.15	2.17	2.61	0.44	0.38	0.18	0.15
		Ktedonobacterales	0.19	0.46	1.20	3.45	0.59	0.59	0.41	2.17	0.65	1.41	0.62	0.91	0.30	0.35	0.28	0.43
Bacteroidetes	Sphingobacteriia	Sphingobacteriales	0.51	0.53	0.67	0.77	1.23	0.50	0.28	0.43	0.64	0.47	0.75	1.02	0.67	0.93	0.50	0.42
	Cytophagia	Cytophagales	0.15	0.13	0.42	1.36	0.22	0.14	0.11	0.31	0.25	0.16	0.21	0.26	0.18	0.20	0.20	0.13
Others			14.70	18.51	10.50	12.08	10.71	12.87	6.30	18.04	11.59	15.00	12.00	12.23	19.29	21.18	14.74	17.25

S, the root surface soil; N, the non-root surface soil.

Table S5. Differences between root surface soil and non-root surface soil in the same sampling area by an analysis of similarities (ANOSIM).

**S soil *vs* N soil**	***P* value**	***R* value**
KGY1	0.2	0.3333
KNY2	0.1	1.0000
KHZ1	0.2	0.2963
KXB1	0.4	0.0741
KXQ2	0.1	0.4444
KHT1	0.1	1.0000
NCX2	0.1	0.6667
NCX3	0.2	0.3333

S, the root surface soil; N, the non-root surface soil.
